# Supplementary material for: Novel 1,2,3-Triazole Derivatives as Mimics of Steroidal System—Synthesis, Crystal Structures Determination, Hirshfeld Surfaces Analysis and Molecular Docking
Source: Molecules. 2021 Jul 2;26(13):4059. doi: 10.3390/molecules26134059 (PMC8271369; doi:10.3390/molecules26134059)
Supplement: Supplementary file 1 [file molecules-26-04059-s001.zip › molecules-1276442-supplementary.pdf]

Electronic Supplementary Information

Novel 1,2,3-triazole derivatives as mimics of steroidal system – synthesis, crystal structures determination, Hirshfeld surfaces analysis and molecular docking

Mateusz Daśko,*[a] Anna Dołęga,[a] Magdalena Siedzielnik,[a] Karol Biernacki,[b] Olga Ciupak,[b] Janusz Rachon,[b] and Sebastian Demkowicz[b]

[a] Department of Inorganic Chemistry, Faculty of Chemistry, Gdańsk University of Technology, Narutowicza 11/12, 80-233 Gdansk, Poland, E-mail: mateusz.dasko@pg.edu.pl.
[b] Department of Organic Chemistry, Faculty of Chemistry, Gdańsk University of Technology, Narutowicza 11/12, 80-233 Gdansk, Poland.

Figure S1........ 2
Figure S2........ 2
Figure S3........ 3
Figure S4........ 3
Figure S5........ 4
Table S1........ 5
Table S2........ 5
Table S3........ 7
Table S4........ 9
Table S5........ 12
Table S6........ 14
Table S7........ 16

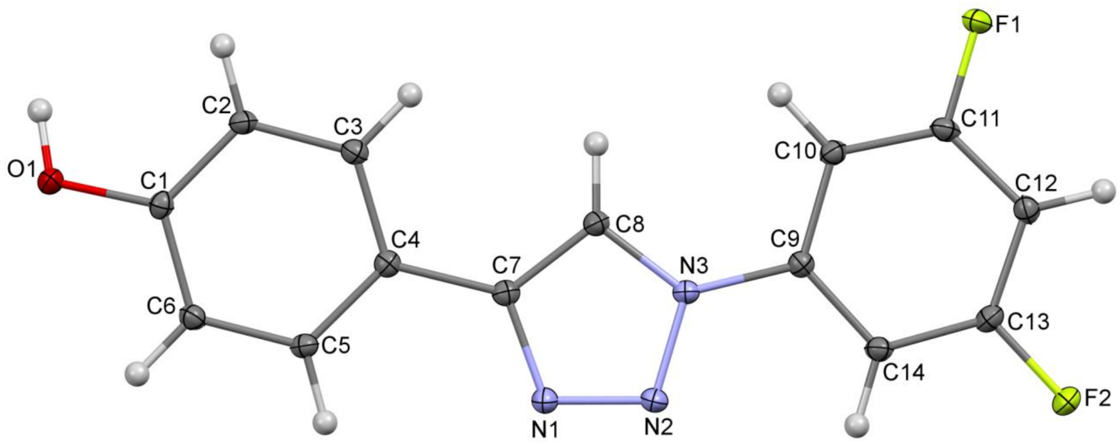

Figure S1. Molecular structure of **6b**. Thermal ellipsoids at 50%.

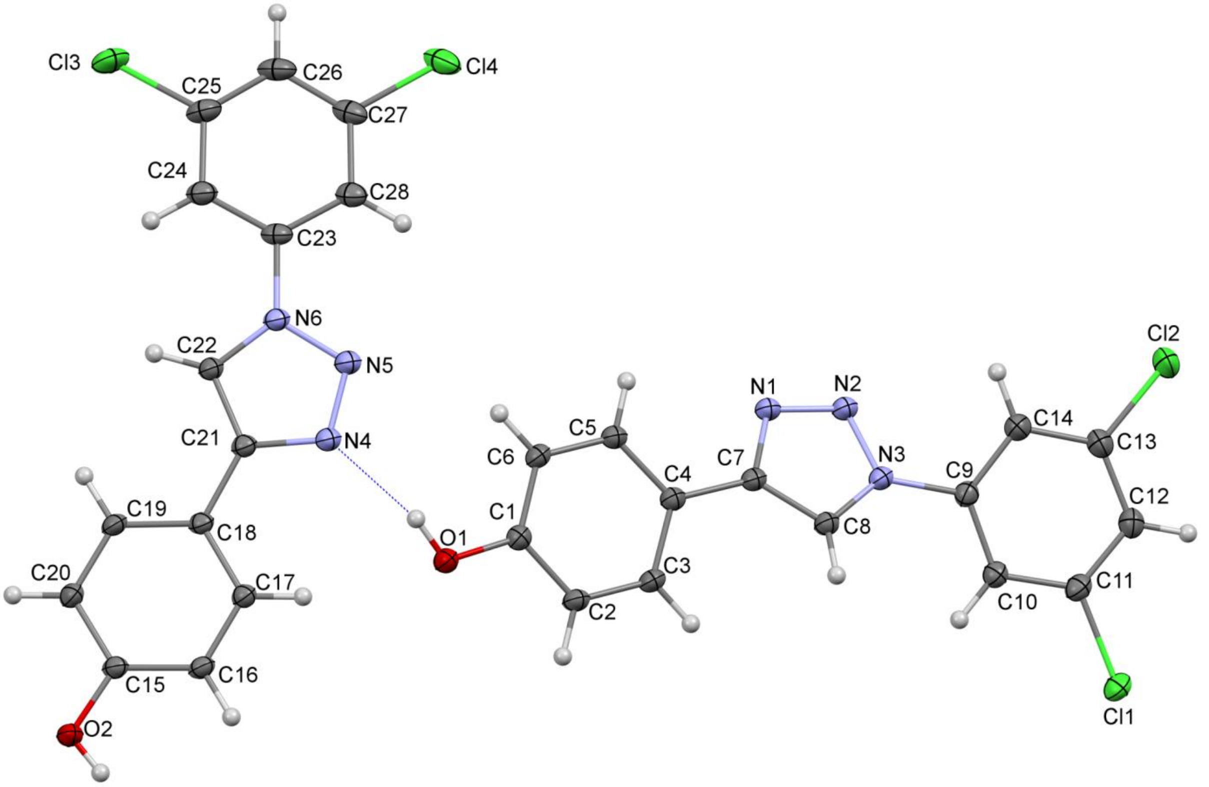

Figure S2. Molecular structures of **6e**. Thermal ellipsoids at 50%.

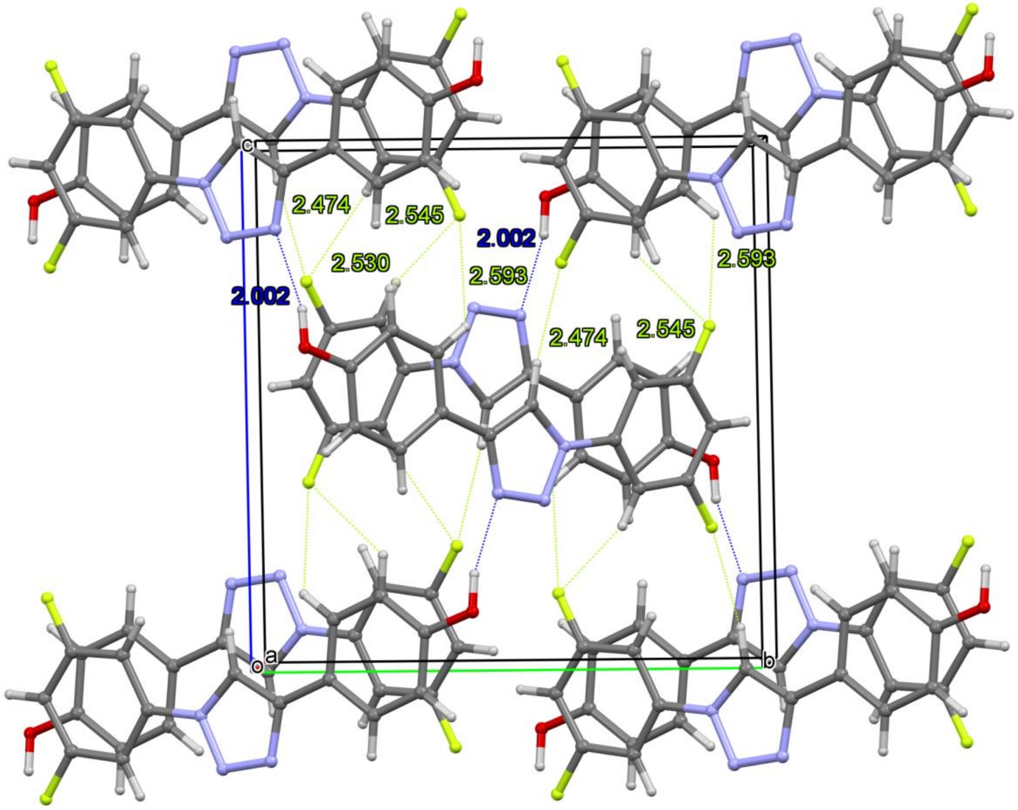

Figure S3. Crystal packings and intermolecular interactions in **6b**.

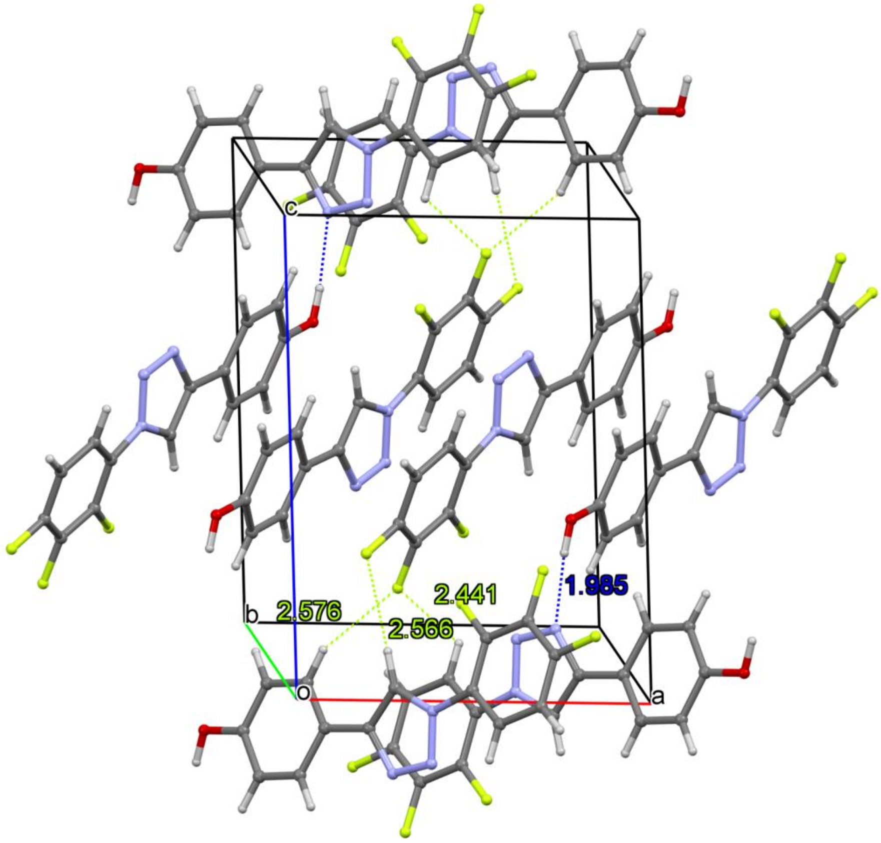

Figure S4. Crystal packings and intermolecular interactions in **6c**.

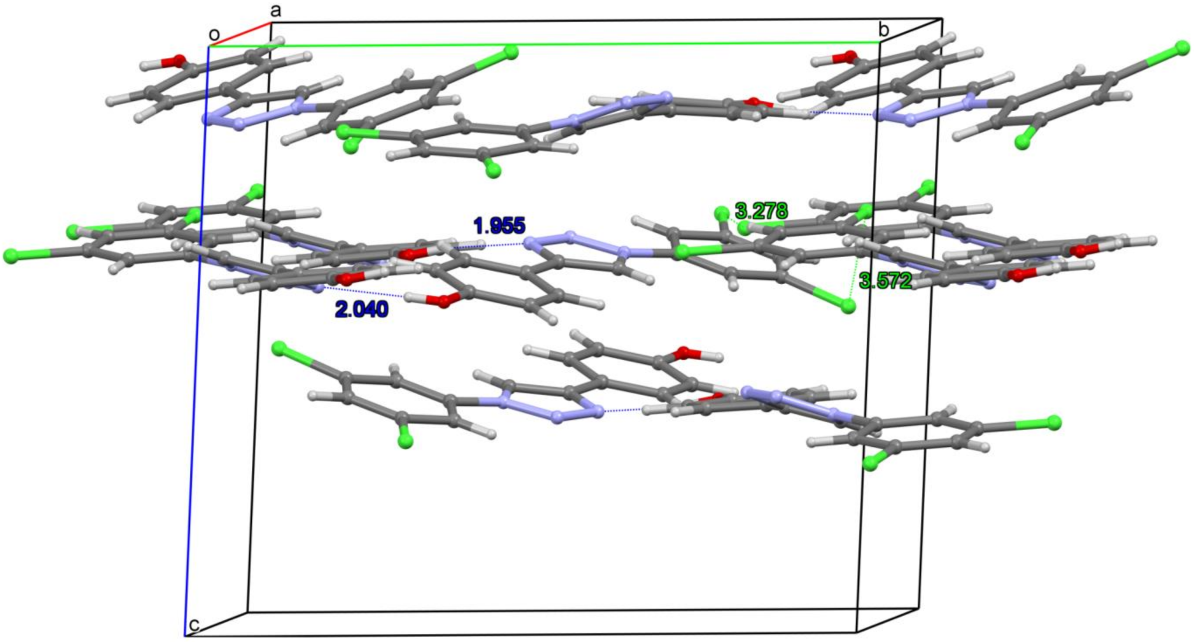

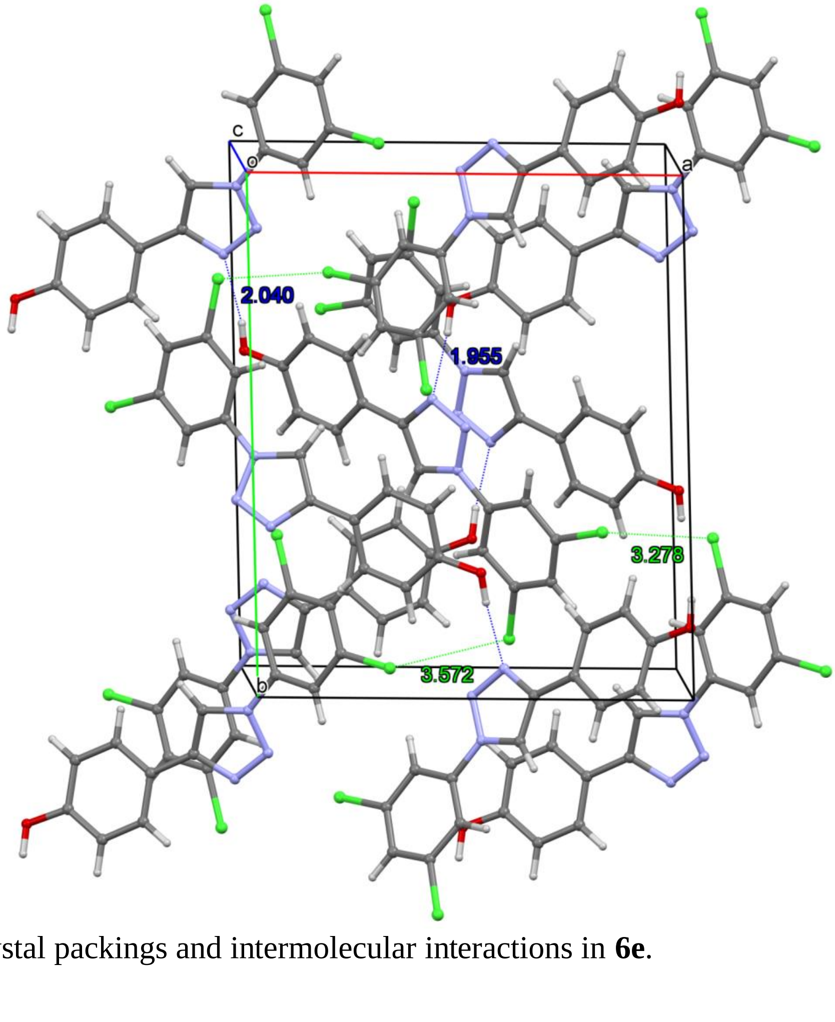

Figure S5. Crystal packings and intermolecular interactions in **6e**.

Table S1. Bond lengths and angles for **6a-e**.

Identification code	**6a**<br>X = F	**6b**	**6c**	**6d**<br>X=Cl	**6e**
Bond lengths [Å]					
Mean C–C in phenol ring(s)	1.390(8)/1.393(5)	1.393(6)	1.392(6)	1.391(4)	1.390(6)/1.390(8)
Mean C–C in halogen substituted ring(s)	1.381(9)/1.384(5)	1.382(6)	1.380(7)	1.387(4)	1.382(8)/1.380(4)
C1–O1/C15–O2	1.360(4)/1.363(3)	1.360(1)	1.366(2)	1.381(2)	1.360(3)/1.359(2)
C4–C7/C18–C21	1.463(4)/1.467(4)	1.467(2)	1.467(2)	1.465(3)	1.465(3)/1.469(3)
C7–N1/C21–N4	1.374(4)/1.373(3)	1.369(1)	1.365(2)	1.374(3)	1.367(3)/1.367(3)
N1–N2/N4–N5	1.316(3)/1.307(3)	1.314(1)	1.308(2)	1.306(2)	1.312(2)/1.315(2)
N2–N3/N5–N6	1.347(3)/1.346(3)	1.351(1)	1.353(2)	1.355(2)	1.348(2)/1.342(2)
N3–C8/N6–C22	1.357(4)/1.355(3)	1.356(1)	1.355(2)	1.358(2)	1.353(3)/1.353(3)
C7–C8/C21–C22	1.356(4)/1.369(4)	1.372(2)	1.373(2)	1.372(3)	1.374(3)/1.371(3)
N3–C9/N6–C23	1.425(4)/1.422(4)	1.423(1)	1.431(2)	1.424(2)	1.432(3)/1.424(3)
C10–F1	-	-	1.345(2)	-	-
C11–X1/X2/C25–F2/Cl3	X1=F1<br>1.358(3)/1.361(3)	X1=F1 1.354(1)	X2=F2 1.340(2)	X1=Cl1 1.740(2)	X1=Cl1<br>1.726(2)/1.736(3)
C12–F3	-	-	1.347(2)	-	-
C13–X2/C27–Cl4	-	X2=F2 1.358(1)	-	-	X2=Cl2<br>1.725(2)/1.724(3)
The torsion angles between the planes of the six-membered rings [°]					
$plane_{C1-C6}$ and $plane_{C9-C14}$	43.28	3.54	3.66	49.35	9.79
$plane_{C15-C20}$ and $plane_{C23-C28}$	50.41	-	-	-	0.90

Table S2. Structural parameters for intramolecular interactions in **6a-e**

**6a**				
Hydrogen bonds				
D-H...A	d(D-H) [Å]	d(H...A) [Å]	d(D...A) [Å]	<(DHA) [°]
O2-H2...N1^{i}	0.97(4)	1.85(4)	2.770(3)	157(3)
O2-H2...N2^{i}	0.97(4)	2.53(4)	3.265(3)	133(3)
O1-H1...N5	1.02(4)	2.55(4)	3.200(3)	122(3)
O1-H1...N4	1.02(4)	1.82(4)	2.758(3)	151(3)
C22-H22...F1^{ii}	0.95	2.34	3.288(3)	173.3
C26-H26...O2^{iii}	0.95	2.57	3.291(4)	132.7
C8-H8...F2^{iv}	0.95	2.52	3.349(3)	146.5
C12-H12...O1^{iv}	0.95	2.45	3.192(4)	134.5
Symmetry transformations used to generate equivalent atoms: ^{i} x, y+1 , z; ^{ii} x, y+1 ,z-1; ^{iii} x, y, z-1; ^{iv} x, y, z+1				
**6b**				
Hydrogen bonds				
D-H...A	d(D-H) [Å]	d(H...A) [Å]	d(D...A) [Å]	<(DHA) [°]
C8-H8...F2^{i}	0.95	2.47	3.4075(16)	167.2
C10-H10...F2^{i}	0.95	2.53	3.4552(15)	164.5
C2-H2...F1^{ii}	0.95	2.55	3.1779(14)	124.2

C12-H12...O1^{iii}	0.95	2.43	3.2566(15)	145.0
O1-H1...N1^{iv}	0.867(19)	2.002(19)	2.8454(15)	164.0(16)
O1-H1...N2^{iv}	0.867(19)	2.648(18)	3.3207(14)	135.3(14)
Symmetry transformations used to generate equivalent atoms: ^{i} x+1/2, -y+3/2, z+1/2; ^{ii} 2 -x+3/2, y-1/2, -z+3/2; ^{iii} x-1, y+1, z; ^{iv} x+1/2, -y+1/2, z+1/2				
**6c**				
Hydrogen bonding				
D-H...A	d(D-H) [Å]	d(H...A) [Å]	d(D...A) [Å]	<(DHA) [°]
O1-H1...N1^{i}	0.87(2)	1.99(2)	2.8514(18)	174.4(18)
C8-H8...F3^{ii}	0.95	2.58	3.5259(19)	178.2
C6-H6...N1^{i}	0.95	2.66	3.348(2)	129.9
C14-H14...F2^{iii}	0.95	2.44	2.9197(19)	111.0
C13-H13...O1^{iv}	0.95	2.53	3.423(2)	156.1
Symmetry transformations used to generate equivalent atoms: ^{i} -x+2, y+1/2, -z+3/2; ^{ii} -x+1, y+1/2, -z+1/2; ^{iii} 3 x, -y+1/2 ,z+1/2; ^{iv} x-1, y-1, z				
**6d**				
Hydrogen bonding				
D-H...A	d(D-H) [Å]	d(H...A) [Å]	d(D...A) [Å]	<(DHA) [°]
O1-H1...O1^{i}	0.78(3)	2.30(3)	3.0651(12)	165(3)
C8-H8...N2^{ii}	0.95	2.68	3.606(3)	163.7
C8-H8...N1^{ii}	0.95	2.63	3.566(3)	167.4
Possible halogen bonding interaction				
Cl1...Cl1^{iii}	3.6618(8)			
Symmetry transformations used to generate equivalent atoms: ^{i} -x+1, y-1/2, -z+1/2; ^{ii} x, y+1, z; ^{iii} 2-x; 1/2+y, 3/2-z				
**6e**				
Hydrogen bonding				
D-H...A	d(D-H) [Å]	d(H...A) [Å]	d(D...A) [Å]	<(DHA) [°]
O2-H2A...N1^{i}	0.78(3)	2.04(3)	2.813(3)	170(3)
O1-H1...N4	0.86(3)	1.96(3)	2.801(3)	167(3)
C16-H16...N2^{i}	0.95	2.66	3.515(3)	150.1
C24-H24...O1^{ii}	0.95	2.57	3.135(3)	118.0
C28-H28...Cl2^{iii}	0.95	2.80	3.698(3)	158.7
Halogen bonding				
Cl4...Cl1^{iv}	3.278(2)			
Symmetry transformations used to generate equivalent atoms: ^{i} x+1, y, z; ^{ii} -x+1,-y+1,-z+1; ^{iii} -x, y-1/2, -z+3/2; ^{iv} x, -1+y, z				

Table S3. The ligand-protein (AROM) interactions (and distances [Å]) identified using BIOVIA, Dassault Systémes, Discovery Studio Visualiser.

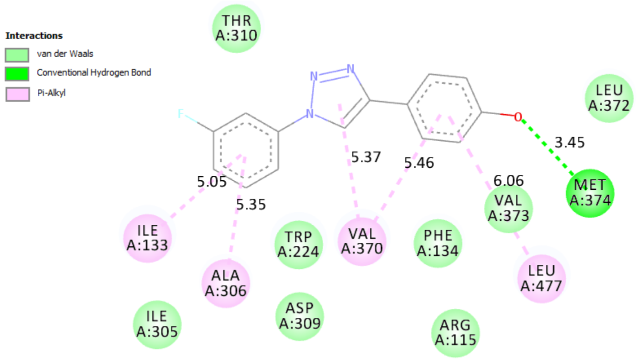

6a

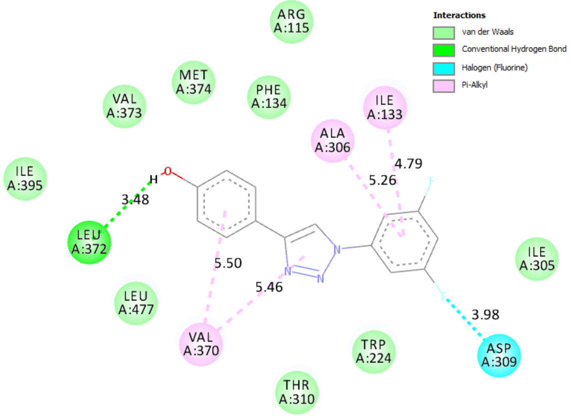

6b

No.	*Type of interaction*							
	van der Waals	*Conventional HB*	*Alkyl*	*π-π-T-shape*	*π-alkyl*	*Carbon HB*	*Sulfur-X*	*Halogen*
6a	ARG115, PHE134, TRP224, ILE305, ASP309, THR310, LEU372, VAL373	MET374 (3.45)	-	-	ILE133 (5.05), ALA306 (5.35), VAL370 (5.37, 5.46), LEU477 (6.06)	-	-	-
6b	ARG115, PHE134, TRP224, ILE305, THR310, VAL373, MET374, ILE395, LEU477	LEU372 (3.48)	-	-	ILE133 (4.79), ALA306 (5.26), VAL370 (5.46, 5.50)	-	-	ASP309 (3.98)
6c	ARG115, PHE134, PHE221, GLU302, THR310, LEU372, VAL373, ILE395	-	-	TRP224 (7.18)	ILE133 (5.22), ALA306 (5.82), VAL370 (5.46), MET374 (6.28), LEU477 (5.81)	ALA306 (2.84)	MET374 (3.29)	ILE305 (3.82), ASP309 (4.12)
6d	ARG115, PHE134, ASP309, THR310, VAL370, LEU372, VAL373, ILE395	-	ILE133 (5.50), TRP224 (4.32), ILE305 (7.01), ALA306 (3.46)	-	ILE133 (5.13), ALA306 (5.87), MET374 (6.05), LEU477 (6.10)	-	MET374 (3.44)	-

6e	PHE221, MET303, ILE305, ASP309, THR310, VAL369, ASP371, VAL373, SER478	GLU302 (4.91)	PHE134 (6.28), VAL370 (5.37), LEU477 (5.01)	TRP224 (6.90)	ILE133 (5.03), ALA306 (5.13), VAL370 (5.01), LEU477 (4.93)	-	-	MET374 (3.74)
Androstenedione	ILE133, PHE134, PHE221, ILE305, ASP309, THR310, LEU372, VAL373, LEU477, SER478	ARG115 (4.05), MET374 (4.03)	TRP224 (7.49), VAL370 (6.51)	-	-	ALA306 (3.36)	-	-

Table S4. The ligand-protein (STS) interactions (and distances [Å]) identified using BIOVIA, Dassault Systémes, Discovery Studio Visualiser.

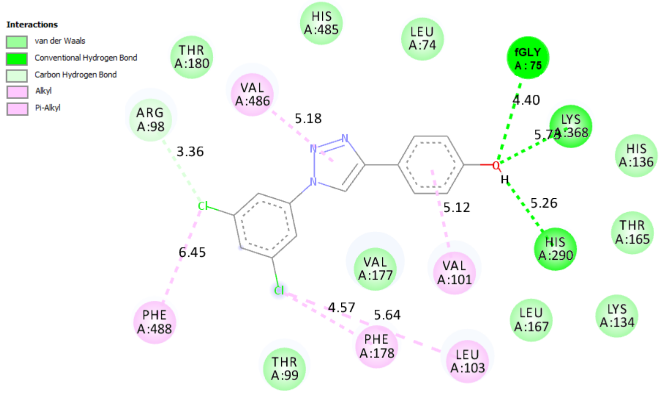

6e

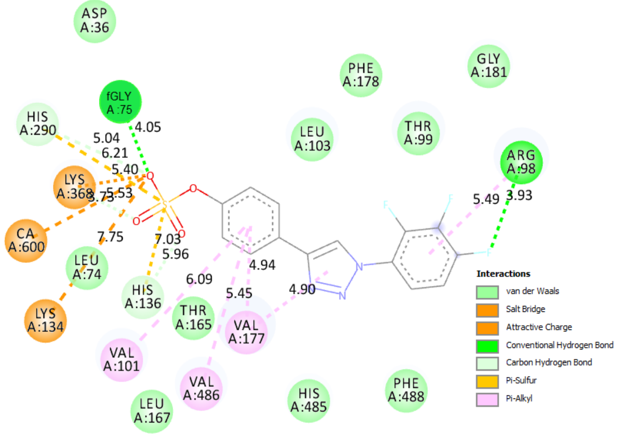

6c*

No.	Type of interaction											
	van der Waals	*Conventional HB*	*π-cation*	*Alkyl*	*π-π-T-shape*	*π-alkyl*	*Carbon HB*	*π-sulfur*	*Salt bridge*	*Attractive charge*	*Halogen*	*Unfavorable donor-donor*
6a	LEU74, THR99, LEU103, LYS134, HIS136, THR165, LEU167, THR180, HIS290, THR484, VAL486	-	LYS368 (6.86)	-	PHE488 (7.37)	VAL101 (5.39), VAL177 (4.87)	HIS485 (5.13)	-	-	-	ARG98 (3.77)	-
6a*	LEU74, THR99, VAL101, LEU103, THR165, LEU167, THR180, THR484, PHE488	fGLY75 (4.21)	-	-	HIS485 (5.02)	ARG98 (5.39), VAL177 (4.76), VAL486 (5.02, 5.20)	HIS136 (6.24), HIS485 (4.47)	HIS290 (6.07), HIS136 (7.23)	-	LYS368 (5.83)	-	-

6b	LEU74, fGLY75, THR99, LEU103, HIS136, LEU167, VAL177, PHE178, THR180, HIS290, PHE488	-	-	-	-	ARG98 (6.05), VAL101 (5.02), VAL486 (5.02)	ARG98 (6.05)	-	-	-	ARG98 (2.96)	LYS368 (4.76)
6b*	LEU74, THR99, LEU103, THR165, LEU167, PHE178, THR180, GLY181, PHE488	fGLY75 (4.07), LYS368 (5.20)	-	-	-	VAL101 (6.15), VAL177 (4.63, 4.66), VAL486 (5.44)	HIS136 (5.66), HIS290 (5.17)	HIS136 (7.10), HIS290 (6.10)	-	LYS134 (7.38), LYS368 (7.45)	ARG98 (3.08)	-
6c	LEU74, LEU103, HIS136, THR165, LEU167, THR180, LYS368, THR484, HIS485, VAL486	fGLY75 (4.37), HIS290 (4.91)	-	-	PHE488 (7.31)	ARG98 (6.36), VAL101 (5.34), VAL177 (4.90)	ARG98 (3.24), THR99 (3.91)	-	-	-	ARG98 (3.55, 5.93)	-
6c*	ASP36, LEU74, THR99, LEU103, THR165, LEU167, PHE178, GLY181, HIS485, PHE488	fGLY75 (4.05)	-	-	-	ARG98 (5.49), VAL101 (6.09), VAL177 (4.90, 4.94), VAL486 (5.45)	HIS136 (5.96), HIS290 (5.04), LYS368 (3.73)	HIS136 (7.03), HIS290 (6.21)	LYS134 (7.75), LYS368 (5.40), Ca^{2+} (5.53)	LYS134 (7.75), LYS368 (5.40), Ca^{2+} (5.53)	-	-
6d	LEU74, THR99, LYS134, HIS136, THR165, LEU167, VAL177, PHE488	fGLY75 (4.31), HIS290 (5.65), LYS368 (5.66)	-	LEU103 (5.89), PHE178 (5.09)	-	ARG98 (6.12), VAL101 (4.97), VAL486 (5.05),	-	-	-	-	-	-

6d*	LEU74, THR99, LEU103, THR165, LEU167, THR180, THR484, HIS485, PHE488	fGLY75 (4.18), LYS368 (5.37)	-	-	-	ARG98 (5.34), VAL101 (6.26), VAL177 (4.84), VAL486 (5.25, 5.41)	HIS136 (6.04)	HIS136 (7.26), HIS290 (6.20)	-	LYS134 (7.93), LYS368 (7.26)	-	-
6e	LEU74, THR99, LYS134, HIS136, THR165, LEU167, VAL177, THR180, HIS485	fGLY75 (4.40), HIS290 (5.26), LYS368 (5.73)	-	LEU103 (5.64), PHE178 (4.57), PHE488 (6.45)	-	VAL101 (5.12), VAL486 (5.18)	ARG98 (3.36)	-	-	-	-	-
6e*	LEU74, THR99, THR165, LEU167, HIS485	fGLY (4.12)	-	ARG98 (3.17), PHE178 (4.47)	-	VAL101 (5.86), LEU103, (6.02) VAL177 (4.86, 4.90), VAL486 (5.84)	ARG98 (3.94), HIS136 (6.08), LYS368 (5.69)	HIS136 (7.03), HIS290 (6.28)	LYS134 (7.88), LYS368 (5.33), Ca^{2+} (3.84)	LYS134 (7.88), LYS368 (5.33), Ca^{2+} (3.84)	-	-
E1	LEU74, ARG98, THR99, GLY100, LYS134, HIS136, THR165, LEU167, VAL486, PHE488	fGLY75 (4.07), HIS290 (5.27)	LYS368 (5.08)	LEU103 (6.83), VAL177 (6.11)	-	VAl101 (4.37)	-	-	-	-	-	-
E1S	ASP36, LEU74, ARG98, THR99, GLY100, LEU103, THR165, THR180, THR484, PHE488	fGLY75 (3.47)	-	HIS485 (7.00), VAL486 (4.89)	-	VAL101 (3.80), VAL486 (6.35)	HIS136 (6.18), HIS290 (5.08)	HIS136 (7.18), HIS290 (5.08)	LYS134 (5.16), LYS368 (5.07), Ca^{2+} (3.47)	LYS134 (5.16), LYS368 (5.07), Ca^{2+} (3.47)	-	-

** in sulfated form*

Table S5. The ligand-protein (17β-HSD1) interactions (and distances [Å]) identified using BIOVIA, Dassault Systémes, Discovery Studio Visualiser.

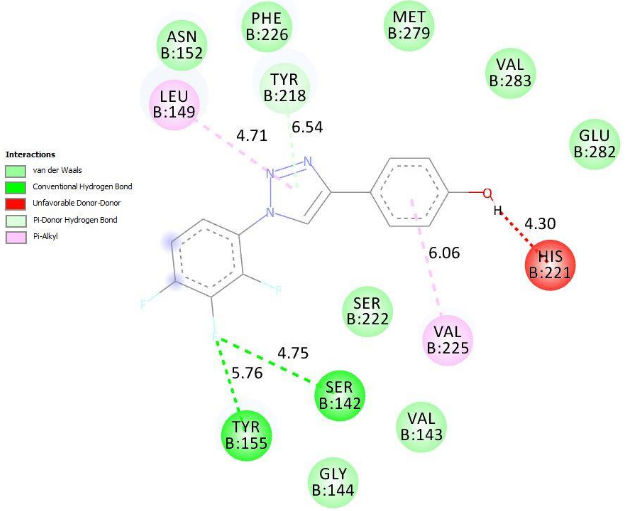

6c

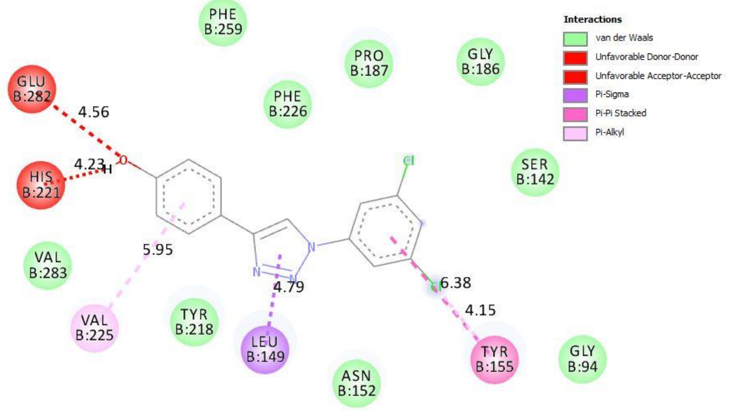

6e

No.	*Type of interaction*										
	van der Waals	*Conventional HB*	*Alkyl*	*π-alkyl*	*Carbon HB*	*Halogen*	*Unfavorable donor-donor*	*π-donor HB*	*π-σ*	*π-π stacked*	*Unfavorable acceptor-acceptor*
6a	PRO187, TYR218, HIS221, PHE226, PHE259, GLU282, VAL283	-	-	VAL225 (5.79)	-	ASN152 (5.73)	-	-	LEU149 (5.01)	TYR155 (6.65)	
6b	ASN152, HIS221, TYR218, PHE226, PHE259, GLU282, VAL283	-	-	VAL225 (5.82)	PRO187 (4.11)	GLY186 (4.58)	-	-	LEU149 (4.91)	TYR155 (6.55)	-

6c	VAL143, GLY144, ASN152, SER222, PHE226, MET279, GLU282, VAL283	SER142 (4.75), TYR155 (5.76)	-	LEU149 (4.71), VAL225 (6.06)	-	-	HIS221 (4.30)	TYR218 (6.54)	-	-	-
6d	GLY94, ASN152, PRO187, TYR218, HIS221, PHE226, PHE259, GLU282, VAL283	-	-	VAL225 (5.86)	-	TYR155 (4.10)	-	-	LEU149 (4.91)	TYR155 (6.56)	-
6e	GLY94, SER142, ASN152, GLY186, PRO187, TYR218, PHE226, PHE259, VAL283	-	-	TYR155 (6.38), VAL225 (5.95)	-	-	HIS221 (4.23)	-	Leu149 (4.79)	TYR155 (6.38)	GLU282 (4.56)
E1	SER142, VAL143, ASN152, TYR155, TYR218, PHE226, ARG258, VAL283	HIS221 (5.20), GLU282 (4.90)	LEU149 (4.60), PRO187 (6.42), PHE259 (7.76)	VAL225 (5.69)	-	-	-	-	-	-	-

Table S6. The ligand-protein (ERα) interactions (and distances [Å]) identified using BIOVIA, Dassault Systémes, Discovery Studio Visualiser.

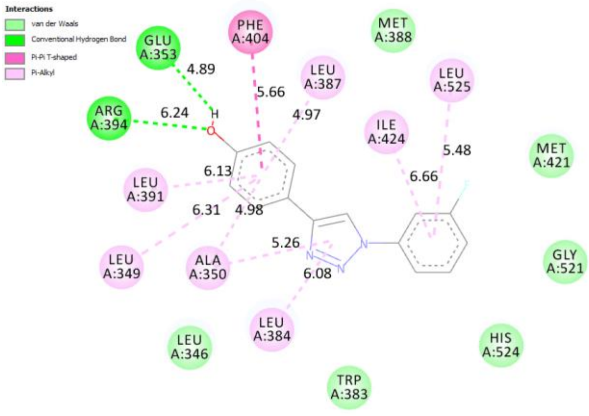

6a

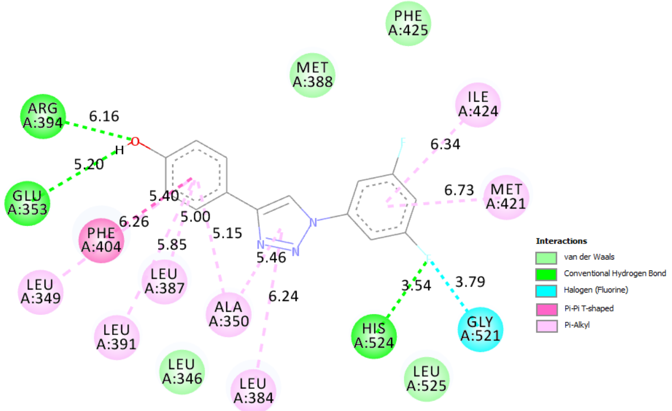

6b

No.	*Type of interaction*								
	van der Waals	*Conventional HB*	*Alkyl*	*π-π-T-shape*	*π-alkyl*	*Carbon HB*	*π-sulfur*	*Halogen*	*π-anion*
6a	LEU346, TRP383, MET388, MET421, GLY521, HIS524	GLU353 (4.89), ARG394 (6.24)	-	PHE404 (5.66)	LEU349 (6.31), ALA350 (4.98, 5.26), LEU384 (6.08), LEU391 (6.13), ILE424 (6.66), LEU525 (5.48)	-	-	-	-
6b	LEU346, MET388, PHE425, LEU525	ARG394 (6.16), GLU353 (5.20), HIS524 (3.54)	-	PHE404 (5.40)	LEU349 (6.26), LEU391 (5.85), LEU387 (5.00), ALA350 (5.15, 5.46), LEU384 (6.24), MET421 (6.73), ILE424 (6.34)	-	-	GLY521 (3.79)	-

6c	GLU353, ALA405, MET421, ILE424, LEU428	ARG394 (6.27), PHE404 (4.44), HIS524 (3.19)	-	PHE404 (5.09)	LEU349 (6.58), ALA350 (5.75), LEU384 (5.68, 6.21), LEU387 (4.85), LEU391 (5.10), LEU525 (6.25)	-	MET388 (5.00)	GLY521 (3.82)	-
6d	LEU428, GLY521	GLU353 (4.54), ARG394 (6.31)	MET421 (4.00), ILE424 (4.16), HIS524 (3.90)	PHE404 (6.12)	LEU349 (6.61), ALA350 (5.70), LEU384 (5.01, 5.18), LEU387 (4.84), LEU391 (5.20), LEU525 (5.99)	-	MET388 (5.53)	-	-
6e	MET343, LEU346, TRP384, ILE424, LEU428	THR347 (3.64), ASP351 (4.54), LYS529 (6.24)	LEU387 (4.28), MET388 (3.53), LEU391 (4.69), PHE404 (6.53), MET421 (5.46), PHE425 (5.78)	PHE404 (3.84)	ALA350 (4.62, 4.62), LEU525 (5.02, 5.21)	LEU384 (4.28)	-	-	ASP351 (6.20)
E2	MET343, LEU346, LEU349, LEU384, MET421, LEU428, GLY521, HIS524, LEU525	GLU353 (4.67), ARG394 (6.52)	MET388 (6.25), PHE404 (6.20), ILE424 (6.92)	PHE404 (5.20)	ALA350 (5.70), LEU387 (5.20), LEU391 (4.64)	-	-	-	-

Table S7. The ligand-protein (ERβ) interactions (and distances [Å]) identified using BIOVIA, Dassault Systémes, Discovery Studio Visualiser.

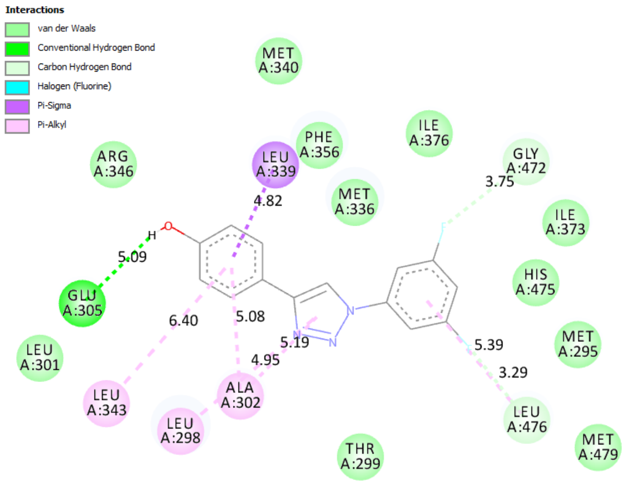

6b

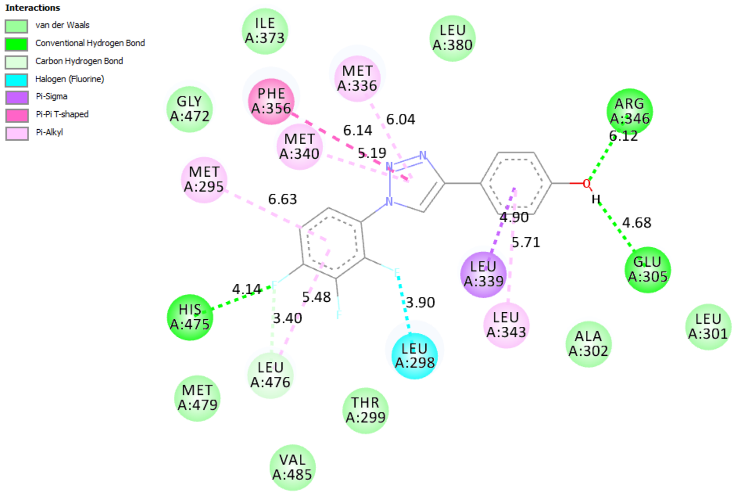

6c

No.	Type of interaction								
	van der Waals	*Conventional HB*	*Alkyl*	*π-π-T-shape*	*π-alkyl*	*Carbon HB*	*π-sulfur*	*Halogen*	*π-Sigma*
6a	LEU301, GLU305, ARG346, PHE356, MET340, LEU298, ILE376, ILE373, HIS475, MET295, LEU490	-	-	-	LEU343 (6.50), ALA302 (5.05, 5.16), LEU476 (5.14)	-	MET336 (5.90)	GLY472 (3.89)	LEU339 (4.63)
6b	LEU301, ARG346, MET340, PHE356, MET336, ILE376, ILE373, HIS475, MET295, MET479, THR299	GLU305 (5.09)	-	-	LEU343 (6.40), ALA302 (4.95, 5.08), LEU298 (5.19), LEU476 (5.39)	LEU476 (3.29), GLY472 (3.75)	-	LEU476 (3.29), GLY472 (3.75)	LEU339 (4.82)

6c	GLY472, ILE373, LEU380, LEU301, ALA302, THR299, VAL485, MET479	ARG346 (6.12), GLU305 (4.68), HIS475 (4.14)	-	PHE356 (6.14)	MET295 (6.63), MET340 (5.19), MET336 (6.04), LEU343 (5.71)	LEU476 (3.40)	-	LEU298 (3.90)	LEU339 (4.90)
6d	LEU301, ARG346, PHE356, MET340, LEU298, GLY472, THR299, LEU490	GLU305 (4.88)	ILE373 (5.09), HIS475 (6.60), ILE376 (4.13)	-	LEU476 (5.05), MET295 (6.60), LEU343 (6.57), ALA302 (4.49, 5.01)	-	MET336 (5.90)	-	MET336 (5.90), LEU339 (4.57)
6e	LEU301, PHE356, MET340, LEU298, GLY472, VAL486, THR299, LEU490	ARG346 (6.02), GLU305 (5.28), LEU339 (3.69)	ILE373 (5.10), ILE376 (3.98), HIS475 (4.14, 5.14), MET479 (4.99), MET295 (4.55), LEU476 (3.36)	-	LEU343 (6.54), ALA302 (4.99, 5.06), LEU476 (5.10)	-	MET336 (5.95)	-	LEU339 (4.62), MET336 (5.95)
E2	GLY472, MET479, LEU476, MET295, LEU298, LEU301, ARG346, MET336, LEU380, ILE373	HIS475 (4.21), GLU305 (4.69)	ILE376 (7.09), MET340 (6.43), PHE356 (4.53)	PHE356 (5.40)	LEU339 (6.07), ALA302 (4.48), LEU343 (6.22)	-	-	-	-
